# Amplicon-based sequencing and co-occurence network analysis reveals notable differences of microbial community structure in healthy and dandruff scalps

**DOI:** 10.1186/s12864-022-08534-4

**Published:** 2022-04-19

**Authors:** Li Wang, Tao Yu, Yaxin Zhu, Yingfeng Luo, Fan Dong, Xuemei Lin, Wenzhong Zhao, Zilong He, Songnian Hu, Zhiyang Dong

**Affiliations:** 1 State Key Laboratory of Microbial Resources, Institute of Microbiology, Chinese Academy of Sciences, No. 1 Beichen West Road, Chaoyang District, Beijing, 100101 China; 2 Department of Microbiology, College of Life Science, State Key Laboratory of Developmental Biology of Freshwater Fish, Hunan Normal University, 36 Lushan Rd., Yuelu District, Changsha, Hunan 410081 China; 3 University of Chinese Academy of Sciences, No.19(A) Yuquan Road, Shijingshan District, 100049 Beijing, China; 4 Lafang China Co.. Ltd., LAF Building, Wanji Industrial Park, Shantou, Guangdong 515041 China; 5 Beijing Advanced Innovation Center for Big Data-Based Precision Medicine, Interdisciplinary Innovation Institute of Medicine and Engineering, Beihang University , No. 37 Xueyuan Road, Haidian District, Beijing, 100191 China

**Keywords:** Scalp, Dandruff, Microbiota, Co-occurrence/co-exclusion network, *Malassezia* sp., *Lactobacillus* sp

## Abstract

**Background:**

Dandruff is a chronic, recurring, and common scalp problem that is caused by several etiopathogeneses with complex mechanisms. Management of this condition is typically achieved via antifungal therapies. However, the precise roles played by microbiota in the development of the condition have not been elucidated. Despite their omnipresence on human scalp little is known about the co-occurrence/co-exclusion network of cutaneous microbiota.

**Results:**

We characterized the scalp and hair surface bacterial and fungal communities of 95 dandruff-afflicted and healthy individuals residing in China. The degree distributions of co-occurrence/co-exclusion network in fungi-bacteria and bacteria-bacteria were higher in the healthy group (*P* < 0.0001), whereas the betweenness values are higher in the dandruff group (*P* < 0.01). Meanwhile, the co-occurrence/co-exclusion network among fungi-fungi and fungi-bacteria showed that compared to the healthy group, the dandruff group had more positive links (*P* < 0.0001). In addition, we observed that *Malassezia slooffiae*, *Malassezia japonica* and *Malassezia furfur*, were more abundant in the dandruff group than in the healthy group. These microbiota were co-exclusion by either multiple bacterial genera or *Malassezia* sp. in healthy group. The lactic acid bacteria on the scalp and hair surface, especially the genera *Lactobacillus* and *Lactococcus*, exhibit a negative correlation with multiple bacterial genera on the scalp and hair surface. *Lactobacillus plantarum* and *Pediococcus lactis* isolated on the healthy human scalp can inhibit the growth of *Staphylococcus epidermidis* in *vitro.*

**Conclusions:**

We showed that microbial networks on scalp and hair surface with dandruff were less integrated than their healthy counterparts, with lower node degree and more positive and stronger links which were deemed to be unstable and may be more susceptible to environmental fluctuations. *Lactobacillus* bacteria have extensive interactions with other bacteria or fungi in the scalp and hair surface micro-ecological network and can be used as targets for improving scalp health.

**Supplementary Information:**

The online version contains supplementary material available at 10.1186/s12864-022-08534-4.

## Background

Dandruff is a skin condition that affects the scalp of up to half the world’s population; this condition is characterized by an itchy, flaky scalp and is associated with various intrinsic and environmental factors, such as sebaceous secretions, skin surface fungal colonization, individual susceptibility [1–3]. Dandruff is believed to be a milder form of seborrheic dermatitis (SD), without visible inflammation and is limited to the scalp [4, 5]. Despite having a high prevalence, the etiology of dandruff has not been fully elucidated. Based on 16S rRNA and ITS gene sequencing of the scalp microbiota, the two most abundant bacterial genera detected on the scalp surface were *Cutibacterium* and *Staphylococcus*, while *Malassezia* was determined to be the predominant fungal inhabitant [6, 7]. Several studies have demonstrated that the formation of dandruff depends on three primary factors: skin surface fungal colonization, sebum secretion and individual susceptibility [8].

Xu et al. speculated that adjusting the balance of scalp bacteria, especially enhancing *Cutibacterium* and suppressing *Staphylococcus* will be a potential solution to lessen dandruff [7]. Bacteria and fungi inhabiting the scalp are known to influence each other and manifestation of dandruff. Patel, C. D. et al. found the interaction of bacterium *Bacillus* sp. strain C2b1 with *Malassezia* sp. strain C2y1 yeast (non-pathogenic) phase of the fungus [9]. Besides, they found that the fungal mycelial surfaces were conducive for interaction with both bacterial cells and yeast forms [9].

It was reported that the bacteriocin produced by *Lactococcus* sp. HY 449 inhibited the growth of *Staphylococcus epidermidis* ATCC 12,228, *Staphylococcus aureus* ATCC 65,389, *Streptococcus pyogenes* ATCC 21,059, and *Cutibacterium acnes* ATCC 6919 [10]. The fermented broth from *Lactococcus lactis* C660 had a growth inhibitory effect on *Staphylococcus epidermidis* that reached of 76% [11]. Novel class II bacteriocins produced by *Lactococcus lactis* BGBU1-4 prevent biofilm formation and/or to eradicate biofilm formed by clinical isolates of coagulase negative *staphylococci* (CoNS) (*Staphylococcus epidermidis*, *Staphylococcus hominis*, *Staphylococcus lugdunensis* and *Staphylococcus haemolyticus*) [12]. The biosurfactant obtained from the probiotic bacterium *Lactococcus lactis* 53 was effective in decreasing the initial deposition rates of *Staphylococcus epidermidis* GB 9/6, *Streptococcus salivarius* GB 24/9 and *Staphylococcus aureus* GB 2/1, allowing for a 90% reduction of the deposition rates [13]. Plantaricins are a group of peptides or small proteins produced by *Lactobacillus plantarum*. Novel plantaricin gene, *pln*1 and *pln*E, which were identified from whole-genome sequencing data of *Lactobacillus plantarum* 163 and heterologous expressed in *Escherichia coli* BL21 (DE3), showed strong antimicrobial activity against gram-positive bacteria such as *Micrococcus luteus* CMCC 63,202, *Staphylococcus epidermidis*, *Lactococcus lactis* NZ3900, *Lactobacillus paracasei* CICC 20,241, and *Listeria innocua* CICC 10,417 [14, 15]. *Staphylococcus epidermidis*, *Streptococcus salivarius*, *Enterococci faecalis*, and lactic acid bacteria (LAB) (*Lactobacillus rhamnosus*, *Lactococcus crispatus*, *Lactococcus lactis*, *Leuconoctoc mesenteroides*), isolated from breast milk of healthy lactating women can inhibit the growth of *Staphylococcus aureus* [16].

The genus *Malassezia* consists of a group of lipophilic yeasts that lack the metabolism necessary for fatty acid synthesis [17]. These fungi are considered to be commensal members of the human skin microbiome [18], where they are predominantly represented by *M. restricta* and *M. globosa*. Recent studies investigating the skin microbiome using culture-free approaches have highlighted the overwhelming dominance of *Malassezia* among eukaryotes on all human body surface sites, especially on the scalp [19, 20]. Eighteen species belonging to the genus *Malassezia* have been isolated to date [21–24]. Developments in the field have helped to elucidate how the development of dandruff is influenced by increased sebum secretion and the proliferation of *Malassezia* [25, 26]. Current anti-dandruff agents primarily have an antimicrobial mode of action, and inhibit growth of *Malassezia* spp. [27]. However, an exact causative relationship between *Malassezia* spp. and dandruff has not been demonstrated due to the high prevalence of *Malassezia* on both healthy and dandruff-affected skin [28, 29]. There is also strong evidence suggesting that individual predispositions and host interactions with *Malassezia*, rather than the mere presence of *Malassezia*, contribute to SD and dandruff pathogenesis [8, 30].

Advances in the characterization of microbial populations and microbiota have enabled a considerably more detailed classification of the microbial composition of dandruff [6, 7, 31–35]. Xu, Z. found that different operational taxonomic units (OTUs) of the same *Malassezia* species exhibited opposing relationships with dandruff, which was consistent with studies conducted on Brazilian and Japanese populations in which different *Malassezia* subtypes were found in different proportions in samples [36, 37]. These findings suggest that not all cases of *Malassezia* are harmful for healthy scalps. The phenomenon that *Malassezia* living on the scalps of healthy people does not cause related diseases indicates that *Malassezia* may be inhibited by other microorganisms*.* In addition, the most abundant bacteria on the scalp (*Cutibacterium* and *Staphylococcus*) showed reciprocal inhibition with each other, which was consistent with the findings of Clavaud and Wang’s works [32, 38]. Compared with a healthy scalp, the dandruff community exhibited decreased *Cutibacterium* and increased *Staphylococcus*, suggesting that the balance between *Cutibacterium* and *Staphylococcus* might be important to the severity of dandruff. In network analysis, the disease groups (dandruff and seborrheic dermatitis) showed lower connectivity and less complex bacterial and fungal networks than did the healthy group [34]. Using bacterial and fungal data of the 204 human skin subjects, Leung MHY, et al. applied correlation networks and found that the majority of the inter-domain associations were positive, central nodes were not necessarily the most abundant OTUs, *Malassezia*, *Cutibacterium* and *Staphylococcus* were involved in both positive and negative cross-domain correlations [39]. Microbial networks on cheeks with acne as well as scalps with dandruff were less integrated than their healthy counterparts, with lower average node degree and decreased network stability upon node attack removal [40, 41].

In our study, using amplicon-based sequencing (16S and ITS1), the scalp and hair surface microbiota of 95 individuals residing in two cities in China (Shantou and Dezhou), including dandruff and healthy individuals, were characterized with a focus on intra- and cross-domain correlation network analysis of the scalp and hair surface microbiota. The present study provides new perspectives for understanding the roles of bacteria and fungi in dandruff scalp and hair surface. We also isolated *Lactobacillus* and *Staphylococcus* bacteria from human scalp and studied their interaction in *vitro*. The results from this study showed that protecting the microbial homeostasis on the scalp is essential for maintaining the health of the scalp.

## Results

### Microbiota profiling of the healthy and dandruff samples

**Fig. 1:**
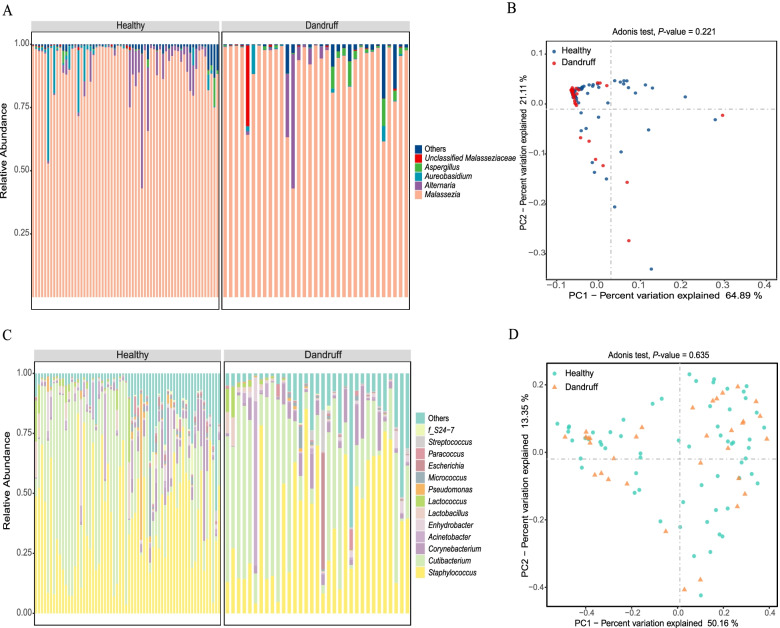
Taxonomic composition and beta diversity of fungi and the bacterial microbiota on the scalp. Fungal (**A**) and bacterial (**C**) genera detected on scalp. Beta diversity based on Bray–Curtis distance of fungi (**B**) and the bacterial (**D**) population

Using the Illumina NovaSeq platform, we obtained 6,259,921 high-quality ITS reads from 95 individuals (Table S1), exhibiting a median of 65,893 reads per sample and a range from 25,074 to 178,031. A total of 1976 OTUs were identified in our study (median = 98 OTUs, ranging from 43 to 516 OTUs in all sample). The 40 OTUs were assigned to the genus *Malassezia* (Table S2). The majority of the fungi community on the scalp and hair surface were *Malassezia* (ranged from 43.0% to 99.5%, median = 96.6%) with 71.0% *Malassezia restricta* (OTU1, identity 100%, ranging from 8.4% to 98.0%, median = 79.0%) (Fig. 1A and Table S3). The remaining 9.2% of sequences were from other non-*Malassezia*. The top four most abundant genera in all samples were *Malassezia*, *Alternaria*, *Aureobasidium*, *Aspergillus* (Fig. 1A). The most predominant fungal species, *Malassezia restricta* (OTU1, 100% identity) was present on the scalp and hair surfaces in both the healthy and dandruff groups and exhibited frequencies of 71.9% and 77.7% of the sequences, respectively. The Principal Coordinate Analyses (PCoA) were based on a Bray–Curtis dissimilarity using evenly sampled OTU abundances. The scalp and hair surface microbiota from healthy and dandruff group did not cluster separately and did not have a significant difference in fungi composition (*P*-value = 0.221) (Fig. 1B). Most subjects between the two different groups overlapped, indicating a similar structure. No significant abundance difference of *Malassezia* at the genus level was detected, although some OTUs, such as *Malassezia slooffiae* (OTU26, 98.3% identity), *Malassezia japonica* (OTU5, 100% identity) and *Malassezia furfur* (OTU10, 100% identity), showed high abundance in the dandruff group (*P* < 0.01 and LDA score > 2) (Figure S1 and Tables S5), while another OTUs, such as *Malassezia sp.* (OTU21), *Malassezia arunalokei* (OTU285, 98% identity) and *Malassezia sp.* (OTU14), showed high abundance in the healthy group (*P* < 0.01 and LDA score > 2) (Figure S1 and Tables S5).

For 16S sequencing, we obtained 7,982,727 high-quality reads from the total bacterial 16S rDNA V4 sequences from 95 individuals. There was an average of 84,028 reads per sample with a range of 41,196 to 227,283 (median = 60,031 ± 1, 118). A total of 5399 OTUs were identified at a threshold of 97% sequence similarity identity (median = 554 OTUs, ranging from 165 to 1128 OTUs per sample). Thirty-three bacterial phyla were detected, but most sequences were assigned to two bacterial phyla: Firmicutes (total = 42.6%, ranging from 4.5% to 85.2%, median = 43.8%) and Actinobacteria (total = 40.8%, ranging from 6.0% to 92.9%, median = 33.7%) (Table S3). Of the 793 identified genera, *Staphylococcus* (total = 38.0%, ranging from 3.6% to 84.5%, median = 35.3%, Firmicutes) and *Cutibacterium* (total = 32.4%, ranging from 1.1% to 92.4%, median = 23.0%, Actinobacteria) comprised more than 70% of the total sequences (Fig. 1C, Table S3). A total of 97.7% OTUs of the *Staphylococcus* was *Staphylococcus* spp., and 92.4% OTUs of the *Cutibacterium* belonged to *Cutibacterium acnes*. Moreover, in the dandruff group, the proportion of the other low-abundance bacteria ranged from 3.2% to 6.4%. The Bray–Curtis index showed that both heathy and dandruff group were not well separated, and did barely have a significant difference in bacteria composition (*P*-value = 0.635, Fig. 1D).

### Overall co-occurrence/co-exclusion network analysis on fungi and bacteria of the scalp and hair surface

**Fig. 2:**
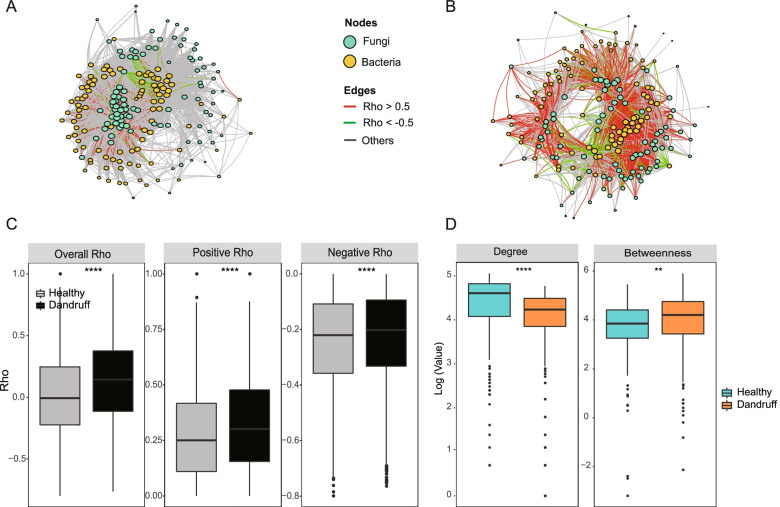
The topological characteristics of fungi-bacteria co-occurrence/co-exclusion networks. **A**, **B** The overall co-occurrence network in healthy and dandruff scalps, respectively. Each node shows one taxa of bacteria or fungi. The size of the node corresponds to the log-transformed degree of the microbiota. The thickness of the edges corresponds to the Spearman’s *r* coefficient (CCREPE). The color of the edges corresponds to the positive (> 0.5) (red) or negative (< -0.5) (green) relationship or weak correlation (-0.5 ≤ *r* ≤ 0.5) (gray). The length of the edges has no meaning. **C** Differences in Spearman’s *r* values between healthy and dandruff scalps at the overall (left), positive (center) and negative (right) levels (****: *P* < 0.0001, Wilcoxon test). **D** Differences in the degree or betweenness of co-occurrence network between healthy and dandruff scalp microbiota (**: *P* ≤ 0.01, ****: *P* < 0.0001, Wilcoxon test)

The topological structure of the overall co-occurrence/co-exclusion network between fungi and bacteria (Fig. 2A and 2B, Table S4 and S6) showed that there were a large number of co-occurrence/co-exclusion links. Compared to the healthy group, the dandruff group exhibited a more stronger correlation (| *r* |> 0.5, spearman correlation calculated by CCREPE) relationships and a lower weaker correlation (| *r* |≤ 0.5, red line means *r* > 0.5, green line means *r* < -0.5, gray line means -0.5 ≤ *r* ≤ 0.5), which suggests networks tend to more unstable and unsteady in dandruff group [39, 42] (Fig. 2B, Table S4). Specifically, the overall correlation and all positive correlations (*r* > 0) were significantly higher in the dandruff group than in the healthy group ( *P* < 0.0001), and all negative correlations (*r* < 0) (Fig. 2C, Table S4) were higher in the dandruff group (*P* < 0.0001), which is more evidence of the instability of the dandruff group network [39, 42].

At the same time, the node-normalized degree (the number of links a node has standardized by the total number of links in the network [43]) of the healthy group's network was significantly higher than the dandruff group (*P* < 0.0001) (Fig. 2D), indicating that the healthy group has more links and is more stable [43]. The dandruff group exhibited significantly higher betweenness centrality (*P* < 0.01) (the number of paths through a node that appears on the shortest path between any other two nodes in the network [43]) values than the healthy group (Fig. 2D), implying that there were more critical “bridge” nodes in the dandruff group, and the loss of these nodes would greatly reduce the stability of the network [43, 44].

**Fig. 3:**
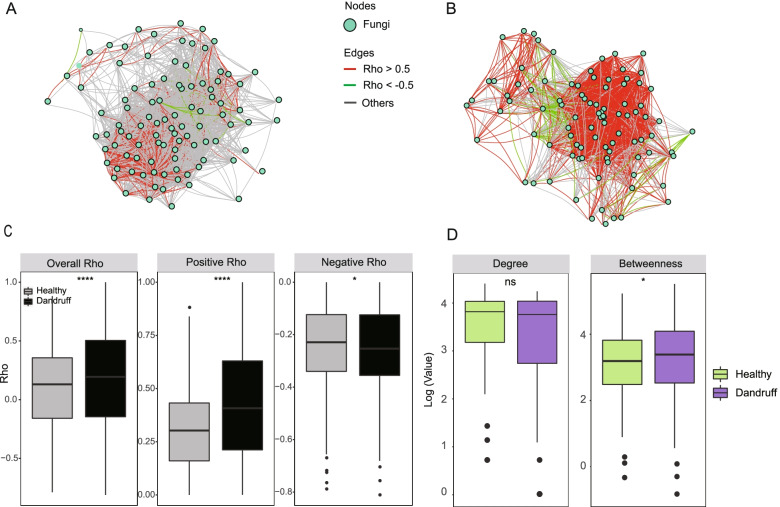
The topological characteristics of fungi-fungi co-occurrence/co-exclusion networks. **A**, **B** The overall co-occurrence network in healthy and dandruff scalps, respectively. The size of the node corresponds to the log-transformed degree of the microbiota. The thickness of the edges corresponds to the | *r* | value of the Spearman relationship. The color of the edges corresponds to the positive (> 0.5) (red) or negative (< -0.5) (green) relationship or weak correlation (-0.5 ≤ *r* ≤ 0.5) (gray). The length of the edges has no meaning. **C** Differences in Spearman’s *r* values between healthy and dandruff scalps at the overall (left), positive (center) and negative (right) levels (*: *P* ≤ 0.05, ****: *P* ≤ 0.0001, Wilcoxon test). **D** Differences in the degree or betweenness of the co-occurrence network between healthy and dandruff scalp microbiota (ns: *P* > 0.05, *: *P* ≤ 0.05, Wilcoxon test)

The results of the co-abundance/co-exclusion network between fungal microbiota (Fig. 3A and 3B) were similar to the results of fungal-bacterial network. The topology of the co-occurrence network showed that compared to the healthy group, the dandruff group has higher stronger correlations (| *r* |> 0.5) and lower weaker correlations (| *r* |≤ 0.5, red line indicates *r* > 0.5, green line indicates *r* < -0.5), gray line indicates -0.5 ≤ *r* ≤ 0.5) (Fig. 3B). Specifically, the overall correlation and all positive correlations (*r* > 0) were significantly higher in the dandruff group than in the healthy group (*P* < 0.0001), and all negative correlations (*r* < 0) (Fig. 3C) were higher in the healthy group (*P* < 0.05), which suggests fungal networks tend to more unstable and unsteady in dandruff group [39, 42]. The slight similarity indicates that the statistical characteristics of the network topology, betweenness distribution of dandruff is significantly higher than healthy groups (Fig. 3D) which is even more suggestive of the instability of the dandruff network.

**Fig. 4:**
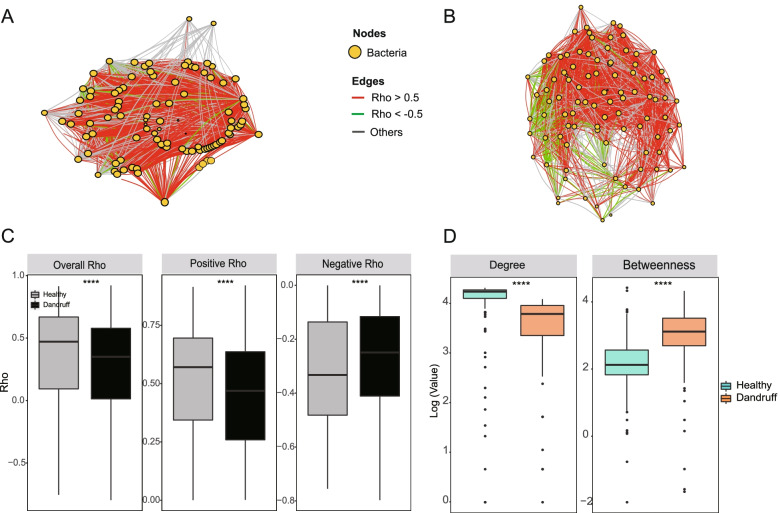
The topological characteristics of bacteria-bacteria co-occurrence/co-exclusion networks. **A**, **B** The overall co-occurrence network in healthy and dandruff scalps, respectively. Each node shows one genus of bacteria. The size of the node corresponds to the log-transformed degree of the microbiota. The thickness of the edges corresponds to the | *r* | value of the Spearman relationship. The color of the edges corresponds to the positive (> 0.5) (red) or negative (< -0.5) (green) relationship or weak correlation (-0.5 ≤ *r* ≤ 0.5) (gray). The length of the edges has no meaning. **C** Differences in Spearman’s *r* values between healthy and dandruff scalps at the overall (left), positive (center) and negative (right) levels (****: *P* ≤ 0.0001, Wilcoxon test). **D** Differences in the degree or betweenness of the co-occurrence network between healthy and dandruff scalp microbiota (****: *P* ≤ 0.0001, Wilcoxon test)

The co-occurrence network of bacteria-bacteria were more complicated. Interestingly, the overall correlation and all positive correlations (*r* > 0) were significantly higher in the healthy group (*P* < 0.0001) (Fig. 4A and 4C). In the dandruff group, all negative correlations (*r* < 0) were higher in the dandruff group (*P* < 0.0001) (Fig. 4B and C), suggesting that bacteria and fungi may have different co-occurrence/co-exclusion relationships and patterns. However, the degree of the bacterial network was higher in the healthy group (*P* < 0.0001), and betweenness was higher in the dandruff group (*P* < 0.0001) (Fig. 4D), which also implies that the dandruff group has a closer interdependence, and it is more likely to be disturbed by the environment and become unstable.

### Highly abundant taxa of co-occurrence/co-exclusion relationships between bacteria and fungi

**Fig. 5:**
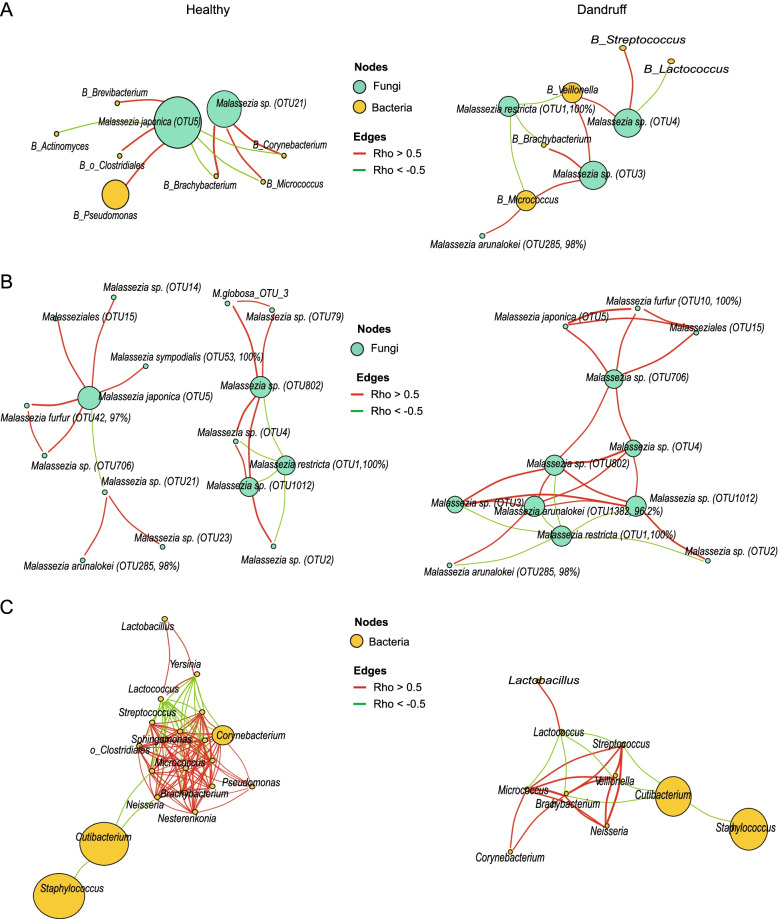
Co-occurrence network between fungi and bacterial microbiota with high abundance on the scalp. **A** fungi-bacteria, (**B**) fungi-fungi and (**C**) bacteria-bacteria, presents healthy scalp on the left side and dandruff scalp on the right side. Each node shows one taxa of bacteria or fungi. The size of the node corresponds to the log-transformed relative abundance of the microbiota. The thickness of the edges corresponds to the | *r* | value of the Spearman relationship (CCREPE). The color of the edges corresponds to positive (> 0.5) (red) or negative (< -0.5) (green)

To detect the network of highly abundant microbiota, the relationships of *P* < 0.05, | *r* |> 0.5 and average abundance greater than 10% were used to filter and reconstruct the network. Analysis of the co-occurrence/co-exclusion relationships between bacteria and fungi in dandruff group showed the following (Fig. 5A): The most abundant fungi, *Malassezia restricta* (OTU1, 100% identity), was negatively related to *Micrococcus*, *Brachybacterium*, and *Veillonella* bacteria. The moderately abundant OTUs of *Malassezia* sp. (OTU3), *Micrococcus*, *Brachybacterium*, *Veillonella*, *Neisseria*, *Sphingomonas* and Methylobacteriacea were positively correlated. More abundant *Malassezia* sp. (OTU4) was positively related to *Streptococcus*, *Veillonella*, *Neisseria*, *Methylobacteriacea* and *Sphingomonas* and negatively correlated with *Lactococcus*.

The *Malassezia* sp. (OTU21) was positively correlated with multiple microbiota, including *Brachybacterium*, *Micrococcus* and *Corynebacterium*. Meanwhile, *Malassezia japonica* (OTU5) was inhibited by multiple bacteria, including *Brevibacterium*, *Clostridiales*, *Pseudomonas*, *Brachybacterium* (Fig. 5A).

The fungi that inhabit the scalp and hair surface of healthy group had significant co-occurrence/co-exclusion relationships and belonged to two clusters (Fig. 5B). The most abundant fungus, *Malassezia restricta* (OTU1, 100%), which was negatively correlated with multiple OTUs of *Malassezia* sp. The other cluster centered on *Malassezia japonica* (OTU5) and *Malassezia* sp. (OTU21), and they inhibited each other. *Malassezia japonica* (OTU5) was positively correlated with *M. sympodialis* (OTU53, 100%), *Malassezia* sp. (OTU14), Malasseziales_OTU15, *M. fufur* (OTU42, 97%), and *Malassezia* sp. (OTU706).

Fungi that inhabit the scalp and hair surface in dandruff group were showed as follows (Fig. 5B right): the most abundant fungus, *Malassezia restricta* (OTU1, 100%), was negatively correlated with *Malassezia arunalokei* (OTU1382, 96.2%) and *Malassezia* sp. (OTU2, OTU3, OTU285, OTU802 and OTU1012). *M. japonica* (OTU5)*, M. furfur* (OTU10, 100%), *Malassezia* sp. (OTU706) and Malasseziales (OTU15) were positively correlated with each other.

The co-occurrence/co-exclusion analysis of the bacterial inhabiting the scalp and hair surface showed that similar to the results of previous studies [37, 39], in healthy group and dandruff-affected group, the two most abundant bacteria *Cutibacterium* and *Staphylococcus* had negative correlations with each other (Fig. 5C). In addition, in the dandruff population *Lactococcus* was negatively correlated with *Micrococcus*, *Brachybacterium*, *Veillonella*, and *Streptococcus*. (Fig. 5C right). The co-occurrence/co-exclusion analysis of scalp and hair surface microbiota in healthy group showed that bacteria, including 14 genera, had a very close positive correlation with each other, while *Lactococcus* had a negative correlation with almost all of these genera (Fig. 5C left). Simultaneously, *Lactobacillus* had a positive correlation with *Lactococcus* (Fig. 5C).

### Taxonomy classification of lactic acid bacteria on the scalp

**Fig. 6:**
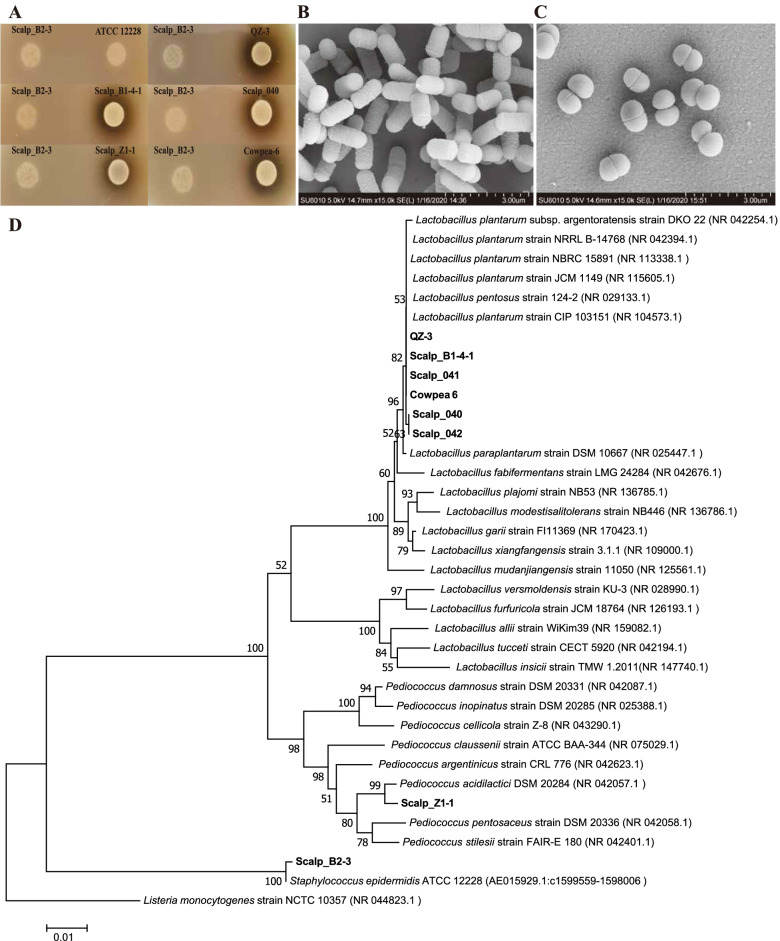
The characterization and antimicrobial activity of *Lactobacillus plantarum* Scalp_B1-4–1, Scalp_040 and *Pediococcus acidilactici* Scalp_Z-1. **A** The inhibition zones of *Lactobacillus plantarum* Scalp_B1-4–1, Scalp_040 and *Pediococcus acidilactici* Scalp_Z1-1. against *Staphylococcus epidermidis* ATCC12228. Scalp_B2-3 isolated from human scalp which was identified as *Staphylococcus epidermidis* was used as the negative control. QZ-3 isolated from silage and Cowpea-6 isolated from pickled cowpeas which were both identified as *Lactobacillus plantarum* were used as the positive control. **B** Scanning electron microscope (SEM) of *Lactobacillus plantarum* Scalp_040. **C** Scanning electron microscope (SEM) of *Pediococcus acidilactici* Scalp_Z1-1 (**D**)The phylogenetic tree based on 16S rRNA gene sequences inferred evolutionary relationships of strain Scalp_B1-4–1, Scalp_040, Scalp_041, Scalp_042, Scalp_Z1-1 by neighbor-joining method

We obtained a series of strains from the scalp cotton swab samples of 5 individuals (B1, B2, D1, G1, E1, Z1) from the Mongolian nationality and 34 individuals living in Shantou city (Table S1) by dilution and spreading on the MRS medium. Scalp_B1-4–1, Scalp_040, Scalp_041, Scalp_042 were identified as *Lactobacillus plantarum* (Fig. 6B), with the 16S rDNA sequence similarity with *Lactobacillus plantarum* strain JCM 1149 was 99.7%, 99.7%, 99.8% and 99.7% (identity) respectively (Fig. 6D, Table S7). Scalp_B2-3 was identified as *Staphylococcus epidermidis*, with the 16S rDNA sequence similarity with *Staphylococcus epidermidis* strain Fussel is 99.8% (Fig. 6D, Table S7). Scalp_Z1-1 was identified as *Pediococcus lactis*, which the 16S rDNA sequence similarity with *Pediococcus acidilactici* DSM 20,284 was 98.5% identity (Fig. 6D, Table S7). These five strains were all isolated from the healthy individuals. The strain QZ-3 isolated from silage has 100% sequence similarity between its 16S rDNA and *Lactobacillus plantarum* strain JCM 1149, and the sequence similarity between the strain Cowpea-6 isolated from pickled cowpea and *Lactobacillus plantarum* strain JCM 1149 is 99.9% (Fig. 6D, Table S7). *Pediococcus acidilactici* Z1-1, which were spherical (0.9–1.1 mm in diameter), Gram-positive, non-spore-forming and appear in pairs, could form opalescent and wet colonies on MRS agar plate (Fig. 6C). Both *Lactobacillus plantarum* B1-4–1, *Lactobacillus plantarum* 040 and *Pediococcus lactis* Z1-1 isolated from the scalp of healthy individuals could inhibit the growth of *Staphylococcus epidermidis* ATCC12228 (Fig. 6A, Figure S2).

## Discussion

Some theoretical studies conjecture that ecological networks composed of weak correlations were more stable than those composed of strong correlations, and the existence of modularity and negative correlations in the network increases the stability of the network under disturbance [39, 42, 45]. Compared to the healthy group, the dandruff group exhibited a stronger correlation (| r |> 0.5) relationships and there was less modularity. The above mentioned results suggest that compared with the relatively stable healthy scalp microbial community, the dandruff group seems to have a closer interdependence and interaction relationship and may be more susceptible to environmental interference. The microbial networks analysis of the bacteria and fungi in our research showed that the average node degree of healthy group was higher than dandruff group. This was consistent to T. Park and Leung results that co-occurrence network of dominant members was a breakdown in dandruff groups [34, 40]. In addition, we also found that the average node betweenness value was higher in the dandruff group in the bacteria-bacteria relationships. Compared to the healthy group, the dandruff group exhibited higher positive links among the fungi-fungi and the fungi-bacteria relationship which was not found in previous studies.

*Malassezia* are considered to be the etiological agents of pityriasis versicolor (PV) and *Malassezia folliculitis*, associated agents in seborrheic dermatitis and contributory factors in other skin disorders, such as atopic dermatitis (AD), psoriasis, confluent and reticulate papillomatosis, and neonatal pustulosis [46]. Although the relative abundance of fungi on human skin was found to be lower than that of bacteria, *Malassezia* yeast were determined to be the most abundant eukaryotes, representing approximately 50–80% of the total skin microbiota [20]. Our research showed that the relative abundance of fungi in *Malassezia* was even higher, reaching 60.2–99.4%. In 79 samples (83%) of 95 samples, the relative abundance of *Malassezia* in fungi exceeded 80%.

The relative abundance of *M. japonica, M. furfur*, and *M. slooffiae* in the dandruff population were significantly higher than in the normal group (*P* < 0.01 and LDA score > 2) (Figure S1 and Tables S5). Previous studies have shown that *Malassezia japonica* is isolated on the skin surface of patients with atopic dermatitis and psoriasis vulgaris [47, 48]. The ratio cultivated in patients is higher than that in healthy people [49]. It has been reported that *M. sympodialis* has been detected in patients with PV, SD [43, 44, 50], and AD [51, 52], and *M. furfur* is the most frequently identified *Malassezia* species associated in the facial lesions of Chinese seborrhoeic dermatitis (SD) in China [53]. Our co-occurence/exclusion network analysis of fungi inhabiting the scalp and hair surface showed that in healthy group, *M. japonica* (OTU5) which was positively correlated with *M. furfur* (OTU42, 97%) and *M. sympodialis* (OTU53, 100%) was negatively correlated with multiple bacterial genera and the highly abundant fungus *Malassezia* sp. (OTU21).

Probiotics as well as resident bacteria can produce antimicrobial peptides that benefit cutaneous immune responses and eliminate pathogens [54]. Previous studies have reported improvements in skin conditions and the prevention of skin diseases by the extracts or ferments of some lactic acid bacteria [55]. Dietary supplementation with β-1,3/1,6-glucan and/or *L. plantarum* LM1004 has considerable potential for the treatment of atopic dermatitis (AD) in humans [56]. It was also reported that certain probiotic preparations show benefit in reducing allergic symptoms in pediatric atopic dermatitis [57]. The cell-free supernatant and protein-rich fraction from *Lactobacillus plantarum* USM8613 inhibited staphyloxanthin biosynthesis, reduced (*P* < 0.05) the cell number of *Staphylococcus aureus* by 10^6^ CFU/mL and reduced biofilm thickness by 55% in *S. aureus*-infected porcine skins [58]. Besides, free and adherent dandruff, erythema and the global clinical score improved significantly (all *P* < 0.05) and restoration of the scalp microbiota enhanced over time in oral application of *Lactobacillus paracasei* NCC 2461 ST11 group compared to the placebo group [59]. In our study, the *L. plantarum* isolated from healthy scalp inhibited the growth of *Staphylococcus epidermidis*.

It was reported that the abundance of *Lactobacilli*, *Burkholderia* spp. and *Cutibacterium acnes* were lower in both atopic dermatitis and psoriasis compared to healthy skin [60]. Healthy skin is normally O_2_ deprived [61]. In patients with atopic dermatitis, due to dry skin and impaired epidermal barrier function [62], oxygenation will increase, leading to a decrease in the abundance of strictly anaerobic bacteria such as *Lactobacillus* spp or *Finegoldia* spp. Under anaerobic conditions, bacteria produce acid by fermenting organic matter, especially forming lactic acid, propionic acid and other short-chain fatty acids (SCFA), which reduces the skin pH to pH < 5.5, thereby protecting the skin from extraneous pathogenic bacteria [60]. We found that *Lactobacillus* and *Lactococcus* on the healthy group have a close negative correlation with multiple bacterial genera (such as *Streptococcus, Micrococcus, Brachybacterium* and *Veillonella*). In the dandruff population, the bacterial genera negatively correlated with *Lactobacillus* and *Lactococcus* decreased significantly. Co-occurrence network analysis showed that the network relationship of microbiota among dandruff group was significantly weaker than healthy group. In dandruff group, both the negative correlation between the potential pathogens of *Malassezia* and other fungi and bacterial genera and the negative correlation between the probiotic lactic acid bacteria and other bacteria were observed to be weaker.

Our research results showed that establishing and protecting the microbial network of the scalp is essential for maintaining the health of the scalp. The occurrence of dandruff, the reduced efficency of antifungal drugs, and the repeated attacks of dandruff are probably related to the destruction of the relationship between scalp microorganisms. The development of hair care products that are conducive to maintaining the relationship between scalp microorganisms will be more helpful to the improvement of dandruff than simply applying a single drug to inhibit *Malassezia*. Research interest in probiotics with nutritive claims, categorized as nutribiotics, has reduced, while interest in therapeutic and pharmacological probiotics, known as pharmabiotics, has recently emerged [63]. So, ointments contain *Lactobacillus plantarum* or its ferment would be helpful to restore microbiota of the scalp. Meantime, it can inhibit growth of some opportunistic pathogen such as *Staphylococcus aureus*. In addition, prebiotics that are beneficial to the proliferation of *Lactobacillus* bacteria are a new direction for the development of anti-dandruff products.

## Conclusions

We characterized the scalp and hair surface microbiota of 95 dandruff-affected and heathy individuals residing in China. The topological structures of the co-occurrence/co-exclusion network between fungi and bacteria showed that the degree values of fungi-bacteria and bacterial co-occurrence networks are higher in the healthy group, whereas the betweenness values are higher in the dandruff group. Besides, we observed that *M. slooffiae*, *M. japonica* and *M. furfur* which can be frequently isolated on the skin surface of patients with seborrhoeic dermatitis, atopic dermatitis and psoriasis vulgaris, were more abundant in the dandruff group than in the healthy group. In addition, in the dandruff population, almost no other fungi or bacteria were found co-exclusion and directly inhibit *M. slooffiae*, *M. japonica* and *M. furfur*. *Lactobacillus plantarum* and *Pediococcus lactis* isolated on the healthy human scalp can inhibit the growth of *Staphylococcus epidermidis*. Our results also showed that protecting the microbial homeostasis on the scalp and hair surface is essential for maintaining the health of the scalp.

## Materials and methods

### Subject recruitment

A total of 95 healthy volunteers with varying dandruff levels were recruited from Shantou and Dezhou, China, aged from18-50 years (Table S1). According to a grading scale as previously described [38], 62 individuals were healthy scalp, and the remaining 33 individuals were dandruff. The study was approved by the Scientific and Ethical Committee in the Institute of Microbiology, Chinese Academy of Sciences (APIMCAS2021146), and was conducted according to the principles expressed in the World Medical Association Declaration of Helsinki. All experiments were performed in accordance with the approved guidelines and regulations. All of the volunteers signed the informed consent, which explained the procedure and purpose of the study. All data were analyzed anonymously, and steps were taken to protect the identities of all participants.

### Sampling of the scalp and hair surface microbiota

Volunteers were advised not to wash their scalp for 2 days before the sampling procedure. The last shampoo was performed two days before the sampling procedure. Samples from the scalp and hair surface were obtained. All operations were carried out in a clean room that had been UV sterilized. The subject’s scalp was washed with 500–1000 mL sterile saline, and the dipping solution was collected into a sterilized basin. A vacuum pump and a suction filter bottle were used to filter the soaking liquid stored in the glass media bottles onto a 0.22-µm microporous filter membrane. If there was more dandruff, filter every 80–100 mL soaking liquid onto a membrane. Each 200 mL of leaching solution was filtered onto a membrane, and the filter membrane containing dandruff (microbes and epidermal cells) was clamped into a sterile 50-mL centrifuge tube with tweezers and stored at -20 °C.

Although the scalp dipping method inevitably introduces the microbial flora attached to the hair surface, this method can obtain the maximum amount of microorganisms on the scalp and hair surface, and reduce the problem of sample inhomogeneity caused by local sampling. In addition, microbial samples obtained by membrane filtration can be stored at -80 ºC for a long time, and DNA extraction and sequencing can be performed multiple times.

### Fungal and bacterial metagenomic DNA extraction

Genomic DNA was extracted from the 0.22 µm microporous filter membrane using the DNeasy PowerWater DNA Isolation Kit from QIAGEN according to the manufacturer's instructions with minor modifications for separate extraction of bacterial and fungal genomic DNA [64].

### PCR amplification and sequencing

Equal concentrations of bacterial and fungal DNA (~ 10 ng) were used for PCR amplification of the bacterial 16S rRNA V4 hypervariable region and fungal ITS1 region. The V4 primers are 515F (5′-GTGCCAGCMGCCGCGGTAA-3′) and 806R (5′-GGACTACHVGGGTWTCTAAT-3′) [65]. For fungi, the ITS1 intergenic region were amplified using the primers ITS1F (5′-CTTGGTCATTTAGAGGAAGTAA-3′) and ITS1R (5′-GCTGCGTTCTTCATCGATGC-3′) [66]. 16S rRNA V4 hypervariable region and ITS1 region were amplified used the specific primer with the barcode. All PCR reactions were carried out in 30 μL reactions with 15 μL of Phusion® High-Fidelity PCR Master Mix (New England Biolabs); 0.2 μM of forward and reverse primers, and about 10 ng template DNA. Thermal cycling consisted of initial denaturation at 98 ºC for 1 min, followed by 30 cycles of denaturation at 98 ºC for 10 s, annealing at 50 ºC for 30 s, and elongation at 72 ºC for 30 s. Finally 72 ºC for 5 min. Mix same volume of 1xloading buffer (contained SYB green) with PCR products and operate electrophoresis on 2% agarose gel for detection. Samples with bright main strip between 400–450 bp were chosen for further experiments. PCR products was mixed in equidensity ratios. Then, mixture PCR products was purified with GeneJET Gel Extraction Kit (Thermo Scientific). Sequencing libraries were generated using Illumina TruSeq DNA PCR-Free Library Preparation Kit (Illumina, USA) following manufacturer’s recommendations and index codes were added. The library quality was assessed on the Qubit^@^ 2.0 Fluorometer (Thermo Scientific) and Agilent Bioanalyzer 2100 system. At last, the library was sequenced on an Illumina NovaSeq platform and 250 bp paired-end reads were generated.

### Bioinformatics and statistical analysis

All sequencing raw data were trimmed using trim-galore (version 0.6.4) [67]. Adaptor sequences, primers and low-quality sequences (quality score < 30, Q30) were trimmed off, and only the reads with a length greater than 150 bp were retained (parameters used: –paired –quality 30 –length 150). The remaining data were processed through the web-based amplicon sequencing data analysis pipeline [68]. In short, paired-end reads were assembled for each amplicon sequence using FLASH [69]. The paired-end joining program and OTU table were generated by UPARSE [70] with a clustering threshold of 0.97. RDP Classifier based on RDP naive Bayesian rRNA Classifier [71] was utilized to assign 16S rRNA or Fungal ITS sequences to the bacterial and fungal taxonomy by RDP training set RDP release 11.5 and unite database (8.2 version 2020–02-04), respectively, with parameter “conf 0.8”. The OTUs classified as chloroplasts and mitochondria were also removed [39]. The alpha diversity was calculated using the Shannon index and observed OTU after rarefying from 1000 sequences at a step size of 5,000 for V4, as well as for ITS1 amplicons, using the Vegan package of R (https://github.com/vegandevs/vegan/). Beta diversity was analyzed by measuring the Bray–Curtis distances for the bacterial and fungal populations at the genus and species level by the Vegan package of R (https://github.com/vegandevs/vegan/). All Principal Coordinate Analyses (PCoA) were based on a Bray–Curtis dissimilarity using evenly sampled OTU abundances. The statistical significance was determined by PERMANOVA with permutations done 999 times using function adonis in the vegan package of R (https://github.com/vegandevs/vegan/). The Linear discriminant Analysis Effect Size (LEfSe) [72] algorithm was utilized to screen for the markedly different OTUs between two groups.

### Co-occurrence/co-exclusion relationships network analysis

To obtain the stable and steady co-occurrence/co-exclusion relationships, OTUs with sample prevalence coverage above 20% were retained to calculate the correlations via Compositionality Corrected by REhealthyization and Permutation (CCREPE version 1.0, R package) with default Spearman correlation similarity measure and iterations of bootstrap and permutation 1000 times [73, 74]. The relationships with a *P* < 0.05 remained to calculate the correlation coefficient distribution with the overall correlation coefficient, positive correlation coefficient and negative correlation coefficient follow the method of Franciska et al. [45]. Wilcoxon tests were used to show the difference between two groups. The network was subsequently calculated and visualized by iGraph [75] of R packages (https://igraph.org/r/). We detected network statistics properties using the connectedness of network nodes by the degree function, using the centrality of network nodes by the betweenness function, and calculated clustering coefficients using the transitivity function packaged in iGraph [75]. To inspect the high abundant taxonomic network, relationships with *P* < 0.05, | *r* |> 0.5 and mean abundances above 1% were utilized to reconstruct the network.

### Isolation of *Lactobacillus plantarum* and determination of its antibacterial activity

Samples from human scalps were collected by rubbing rayon swabs (plain swab, sterile; REF70610, bioMerieux, France) on the scalp. Then soak the cotton swab in 1.5–2 mL of sterile saline (0.9% NaCl solution) and place it in a 1.5–2 mL EP tube, sealed and stored at 4 °C. Then the cotton swab soaking solution was taken from the original solution, 1/10 dilution, 1/100 dilution and 100 µl dilutions was spread on Man Ragosa Sharpe (MRS) agar plates (Peptone10 g/l, Meat extract 8 g/l, Yeast extract 4 g/l, Glucose 20 g/l, CH_3_COONa·3H_2_O 5 g/l, Tween 80 1 g/l, Dipotassium hydrogen phosphate 2 g/l, Triammonium citrate 2 g/l, MgSO_4_·7H_2_O 0.2 g/l, MnSO_4_·4H_2_O 0.05 g/l, Agar 10 g/l, pH 6.2, 25 °C), placed in an anaerobic jar (Oxoid AG0025A 2.5 l), sealed and incubated anaerobic at 37 °C for 3 days. The colonies on the solid plate were inoculated into a test tube containing 2 ml of MRS liquid medium, placed in an anaerobic jar, sealed and cultured anaerobic at 37 °C for 24–48 h. Centrifuge part of the bacterial solution at 12,000 rpm, take the extracellular fermentation broth for pH determination, extract the total bacterial DNA and identify the 16S rDNA for the strains with pH value of the fermentation broth < 5.0, and add the bacterial solution to the final concentration of 20% glycerol, -80 °C Freeze storage.

The Scalp_B1-4–1, Scalp_040 and Scalp_Z-1–1 were grown on MRS liquid medium at 37 °C for 24 h under anaerobic condition, and then scanning electron microscopy (SEM, Hitachi SU8010, Japan) were used to observe the morphology of the bacterial cell. The QZ-3 (isolated from silage) and Cowpea-6 (isolated from pickled cowpea) were grown on MRS liquid medium at 37 °C for 24 h under anaerobic conditions. The Scalp_B2-3 and standard strain *Staphylococcus epidermidis* ATCC12228 (Purchased from CGMCC, China General Microbiological Culture Collection Center, CGMCC 1.4260) were grown on MRS liquid medium at 37 °C for 48 h under anaerobic conditions. Biochemical characterization of Scalp_B1-4–1, Scalp_040 and Scalp_Z-1–1 was done subsequently. Genomic DNA was extracted from these strains using a genomic DNA extraction kit according to the manufacturer’s instructions (Sangon, China). The partial 16S rRNA gene sequence was determined from these strains using primers 27F (5’-AGAGTTTGATCCTGGCTCAG-3’) and 1492R (5’-CGGTTACCTTGTTACGACTT-3’) (Wu et al., 2010; Yi et al., 2018), or Lac16S-for (5’-AATGAGAGTTTGATCCTGGCT-3’) and Lac16S-rev (5’-GAGGTGATCCAGCCGCAGGTT-3’) [75]. The reaction mixture (20 µl) contained 30 ng template DNA, 1.5 mM MgCl_2_, 0.2 mM dNTPs, 1 µM each primer and 1 U Taq DNA polymerase (TransStart® FastPfu DNA Polymerase) in a standard reaction buffer. After an initial denaturation of 4 min at 94 °C, 25 cycles of 1 min at 94 °C, 1.5 min at 50 °C, 2 min at 72 °C and a final extension at 72 °C for 7 min were performed. The 1.6 kb amplification product was extracted from agarose gel (TIANgel Midi Purification Kit, TIANGEN DP209-03) and sequenced at BGI-Beijing. The sequence obtained was aligned with the 16S rRNA gene sequences of other *Lactobacillus* and *Pediococcus* using the CLUSTAL_X program [72]. The phylogenetic tree was constructed on the basis of 16S rRNA genes by the neighbor-joining method using MEGA7.0.26 software and evolutionary distances were computed using the maximum composite likelihood method [76].

The *Lactobacillus plantarum* strains above isolated from healthy individual scalp were cultured in MRS broth medium in an anaerobic jar at 37 °C for 24 h, and 3 µl were inoculated on MRS agar plates and cultured anaerobic at 37 °C for 24 h. The *Lactobacillus plantarum* QZ-3 isolated from silage and the *Lactobacillus plantarum* Cowpea-6 isolated from pickled cowpea were used as the positive control, and *Staphylococcus epidermidis* B2-3 isolated from the scalp of healthy individuals and the standard strain *Staphylococcus epidermidis* ATCC12228 were used as the negative control. Besides, *Staphylococcus epidermidis* ATCC12228 was also used as the indicator bacteria, it was cultured in MRS broth medium in an anaerobic jar at 37 °C for 48 h until the number of bacterial cells reaches 10 x 10^12^ cell/ml. The inoculation ratio of 1 ml bacterial liquid to 100 ml melted MRS solid medium was used as the upper layer and was anaerobic cultured at 37 °C for 24-48 h. Then, the transparent zone was measured.

## Supplementary Information

**Additional file 1:** **Figure S1.** The markedly different taxa between healthy and dandruff groups via LEfSe analysis.

**Additional file 2:** **Figure S2.** The inhibition zones of *Lactobacillus plantarum* Scalp_B1-4-1, Scalp_040 and *Pediococcus acidilactici* Scalp_Z1-1. against *Staphylococcus epidermidis* ATCC12228.

**Additional file 3:** **Table S1.** The 95 individual metadata and baseline characteristics. **Table S2.** The result of *Malassezia sp*. identified in this study by RDP classifier compared to NCBI ITS fungi database using blast. **Table S3.** The abundance of fungi and bacteria with at least appeared in 20% samples. **Table S4.** The Spearman correlation coefficient (CCREPE) of different co-occurrence/co-exclusion networks. **Table S5.** The markedly different genera between healthy and dandruff groups by Linear discriminant Analysis Effect Size (LEfSe). **Table S6.** The spearman correlation calculated by SparCC with 1000 bootstraps. **Table S7.** The nearly 1.6 kb 16S rRNA gene annotation with *Lactobacillus* and *Pediococcus* in NCBI nr database via the CLUSTAL_X program.

## Data Availability

Raw sequencing data are available in National Microbiology Data Center under study accession number NMDC10017716 (https://nmdc.cn/resource/genomics/project/detail/NMDC10017716).

## Abbreviations

OTU: Operational taxonomic unit
ITS: Internally transcribed spacer
PV: Pityriasis versicolor
SD: Seborrheic dermatitis
AD: Atopic dermatitis
