# Supplementary figures and images for: Amplicon-based sequencing and co-occurence network analysis reveals notable differences of microbial community structure in healthy and dandruff scalps
Source: BMC Genomics. 2022 Apr 19;23:312. doi: 10.1186/s12864-022-08534-4 (PMC9017024; doi:10.1186/s12864-022-08534-4)

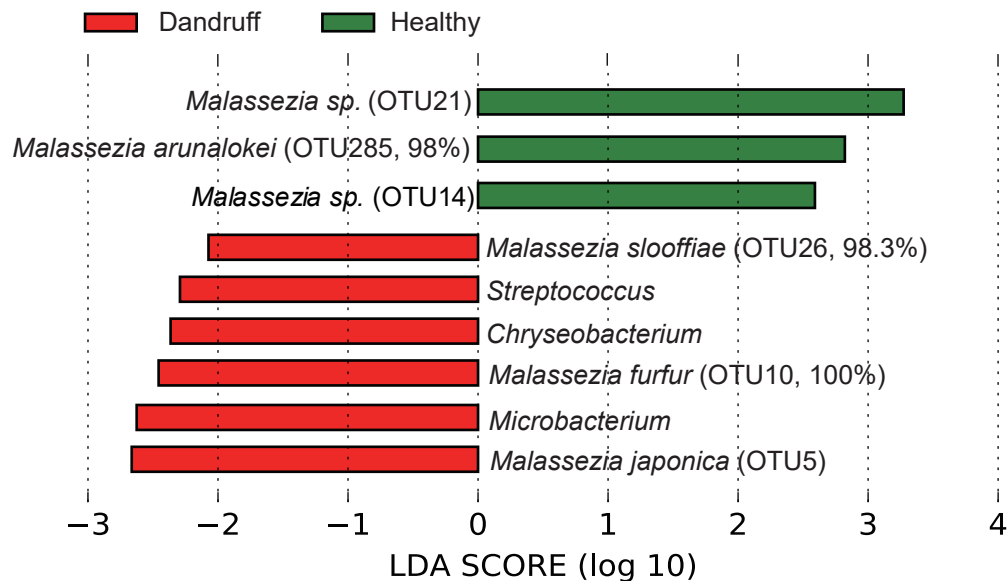

Supplement: Supplementary file 1 — Additional file 1: Figure S1. The markedly different taxa between healthy and dandruff groups via LEfSe analysis. [file 12864_2022_8534_MOESM1_ESM.pdf]

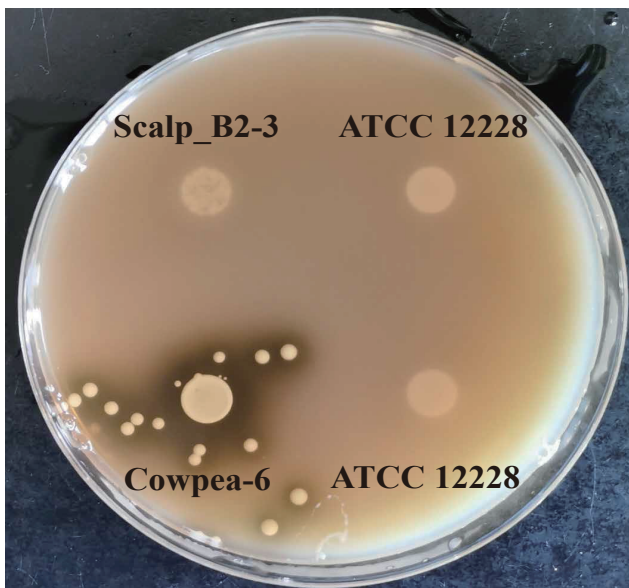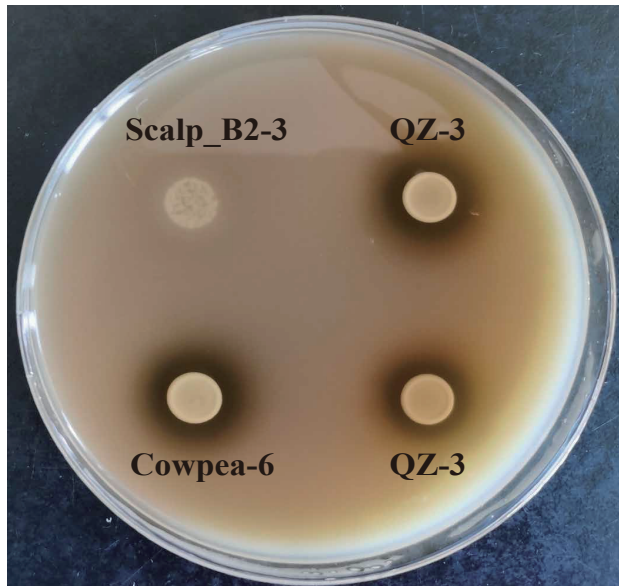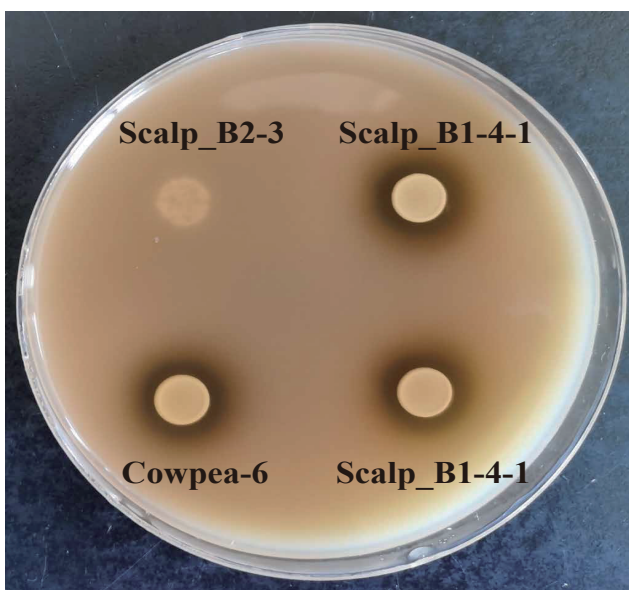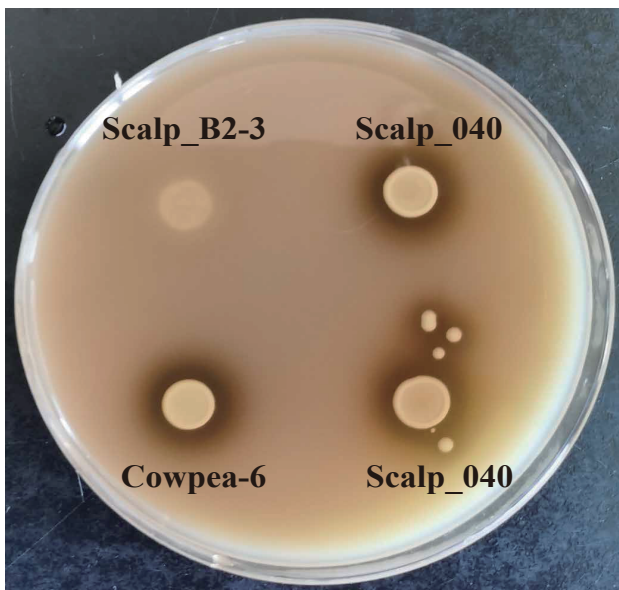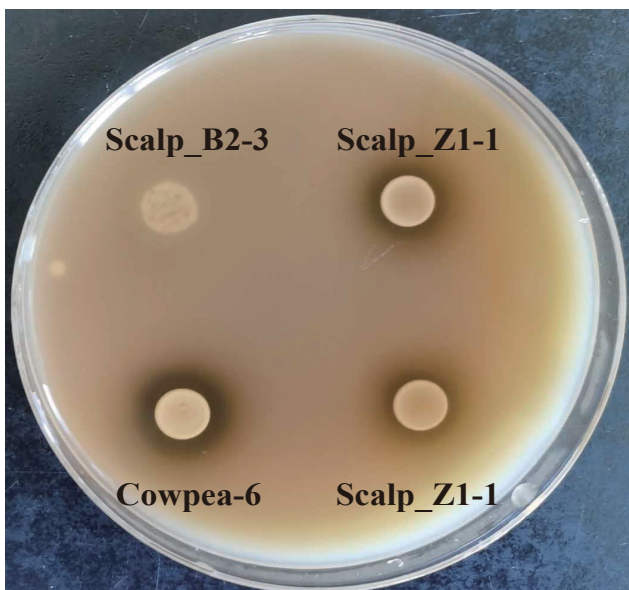

Supplement: Supplementary file 2 — Additional file 2: Figure S2. The inhibition zones of Lactobacillus plantarum Scalp_B1-4-1, Scalp_040 and Pediococcus acidilactici Scalp_Z1-1. against Staphylococcus epidermidis ATCC12228. [file 12864_2022_8534_MOESM2_ESM.pdf]
